# (*E*)-3-Propoxymethyl­idene-2,3-dihydro-1*H*-pyrrolo[2,1-*b*]quinazolin-9-one monohydrate

**DOI:** 10.1107/S1600536810014303

**Published:** 2010-04-30

**Authors:** Burkhon Zh Elmuradov, Kambarali Turgunov, Bakhodir Tashkhodjaev, Khusniddin M. Shakhidoyatov

**Affiliations:** a S.Yunusov Institute of the Chemistry of Plant Substances, Academy of Sciences of Uzbekistan, Mirzo Ulugbek Str. 77, Tashkent 100170, Uzbekistan

## Abstract

The title compound, C_15_H_16_N_2_O_2_·H_2_O, was synthesized *via* the alkyl­ation of 3-hydroxy­methyl­idene-2,3-dihydro-1*H*-pyrrolo[2,1-*b*]quinazolin-9-one with *n*-propyl iodide in the presence of sodium hydroxide. The organic mol­ecule and the water mol­ecule both lie on a crystallographic mirror plane. In the crystal structure, inter­molecular O—H⋯O and O—H⋯N hydrogen bonds link the components into extended chains along [100].

## Related literature

For the synthesis of the title compound and its derivatives, see: Späth & Platzer, (1935 ▶); Shakhidoyatov *et al.* (1976 ▶); Oripov *et al.* (1979 ▶); Elmuradov *et al.* (2006 ▶); Elmuradov & Shakhidoyatov (2004 ▶); Jahng *et al.* (2008 ▶). For the physiological activity of the title compound and its derivatives, see: Amin & Mehta (1959 ▶); Chatterjee & Ganguly (1968 ▶); Yakhontov *et al.* (1977 ▶); Yunusov *et al.* (1978 ▶); Johne (1981 ▶); Shakhidoyatov (1988 ▶). For standard bond distances, see: Allen *et al.* (1987 ▶).
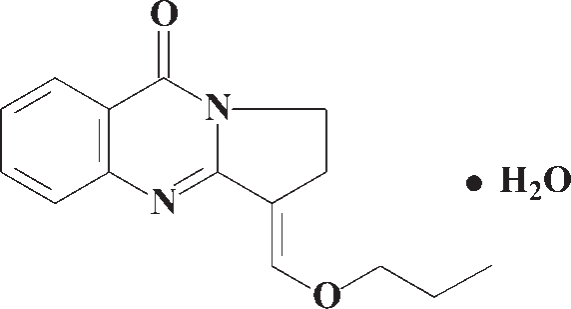


## Experimental

### 

#### Crystal data

C_15_H_16_N_2_O_2_·H_2_O
*M* *_r_* = 274.31
Monoclinic, 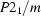

*a* = 9.247 (2) Å
*b* = 6.876 (1) Å
*c* = 10.950 (2) Å
β = 97.90 (3)°
*V* = 689.6 (2) Å^3^
*Z* = 2
Mo *K*α radiation
μ = 0.09 mm^−1^
*T* = 293 K
0.75 × 0.53 × 0.20 mm

#### Data collection

Stoe Stadi-4 four-circle diffractometer
1483 measured reflections
1479 independent reflections
1000 reflections with *I* > 2σ(*I*)
3 standard reflections every 60 min  intensity decay: 2%

#### Refinement

*R*[*F* ^2^ > 2σ(*F* ^2^)] = 0.064
*wR*(*F* ^2^) = 0.144
*S* = 1.15
1479 reflections
128 parameters
H atoms treated by a mixture of independent and constrained refinement
Δρ_max_ = 0.21 e Å^−3^
Δρ_min_ = −0.19 e Å^−3^

### 

Data collection: *STADI4* (Stoe & Cie, 1997 ▶); cell refinement: *STADI4*; data reduction: *X-RED* (Stoe & Cie, 1997 ▶); program(s) used to solve structure: *SHELXS97* (Sheldrick, 2008 ▶); program(s) used to refine structure: *SHELXL97* (Sheldrick, 2008 ▶); molecular graphics: *XP* (Bruker, 1998 ▶); software used to prepare material for publication: *SHELXL97*.

## Supplementary Material

Crystal structure: contains datablocks I, global. DOI: 10.1107/S1600536810014303/lh5028sup1.cif

Structure factors: contains datablocks I. DOI: 10.1107/S1600536810014303/lh5028Isup2.hkl

Additional supplementary materials:  crystallographic information; 3D view; checkCIF report

## Figures and Tables

**Table 1:** Hydrogen-bond geometry (Å, °)

*D*—H⋯*A*	*D*—H	H⋯*A*	*D*⋯*A*	*D*—H⋯*A*
O1w—H1⋯N1	0.85 (7)	2.14 (8)	2.968 (5)	165 (7)
O1w—H2⋯O2^i^	0.90 (7)	1.95 (7)	2.855 (5)	176 (6)

Symmetry code: (i) 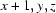.

**Fig. 1.**
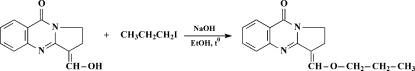
The reaction scheme.

**Fig. 2.**
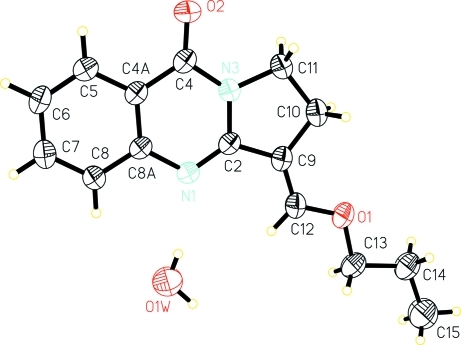
The molecular structure of (I), with atom labels and 50% probability displacement ellipsoids for non-H atoms.

**Fig. 3.**
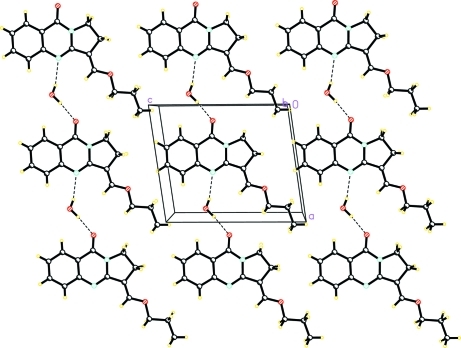
Part of the crystal structure of (I), showing the formation of hydrogen-bonded (dashed lines) chains along [100].

## supplementary crystallographic information

### Comment

Tricyclic quinazoline alkaloids are a large group of heterocyclic compounds (Späth & Platzer, 1935; Shakhidoyatov *et al.*, 1976; Oripov *et al.*, 1979; Elmuradov & Shakhidoyatov, 2006; Jahng *et al.*, 2008). These compounds and their derivatives possess different pharmacological activities (Amin & Mehta1959; Chatterjee & Ganguly, 1968; Yakhontov *et al.*, 1977; Yunusov *et al.*, 1978; Johne, 1981; Shakhidoyatov, 1988).

Alkylation of 3- hydroxymethylidene-2,3-dihydro-1*H*-pyrrolo[2,1-*b*]quinazolin-9-one with C1—C3 alkyl halides leads to the formation of new *C*-alkyl (Elmuradov & Shakhidoyatov, 2004) or *O*-alkyl derivatives (Elmuradov *et al.*, 2006). Using the typical synthesis for *O*-alkyl derivatives the reaction of 3- hydroxymethylidene-2,3-dihydro-1*H*-pyrrolo[2,1-*b*]quinazolin-9-one with n-propyl iodide was carried out by boiling of the initial reagents (1:2 ratio) over 7 hours in ethanol in the presence of sodium hydroxide (Elmuradov *et al.*, 2006) (Figure 1). The compound (3-hydroxymethylidene-2,3-dihydro-1*H*-pyrrolo[2,1-*b*]quinazolin- 9-one) is obtained by formylation of 2,3-dihydro-1*H*-pyrrolo[2,1-*b*]quinazolin-9-one (alkaloid Deoxyvasicinone, isolated from *Peganum Harmala)* (Chatterjee & Ganguly, 1968) with Vilsmeier-Haack reagent.

The asymmetric unit contains half molecule of 3-propoxymethylidene-2,3-dihydro-1*H*-pyrrolo[2,1-*b*]quinazolin-9- one and a half water molecule (Figure 2). Both molecules of the asymetric unit lay on the crystallographic mirror plane. The water molecule links the N and O^i^ atoms of title compound molecule by O—H···N and O—H···O^i^ hydrogen bonds, which form a H-bond chain along [100] (Figure 3). The bond distances (Allen *et al.*, 1987) and angles in molecule are in normal ranges.

### Experimental

Sodium hydroxide (0.08 g, 2 mmol) was dissolved in ethanol (10 ml), and 3-hydroxymethylidene-2,3-dihydro-1*H*-pyrrolo[2,1-*b*]quinazolin-9-one (0.214 g, 1 mmol) and propyl iodide (0.34 g, 0.18 ml, d=1.747 g/ml, 2 mmol) were added. The mixture was heated to reflux on a water bath for 7 hours. The solvent was distilled off and the residue was re-crystallized from hexane. The title compound was obtained in 70 % yield (0.18 g). Colorless crystals suitable for X-ray analysis were obtained from hexane by slow evaporation.

### Refinement

Carbon-bound H atoms were positioned geometrically and treated as riding on their C atoms, with C—H distances of 0.93 Å (aromatic) and 0.97 Å (CH_2_) and 0.96 Å (CH_3_) and were refined with U_iso_(H) =1.2U_eq_(C)]. The H atoms of the water molecule involved in the intramolecular hydrogen bonds were located by difference Fourier synthesis and refined freely [O—H = 0.84 (7) and 0.90 (7) Å].

### Crystal data

**Table:**

C_15_H_16_N_2_O_2_·H_2_O	*F*(000) = 292
*M**_r_* = 274.31	*D*_x_ = 1.321 Mg m^−^^3^
Monoclinic, *P*2_1_/*m*	Mo *K*α radiation, λ = 0.71073 Å
Hall symbol: -P 2yb	Cell parameters from 15 reflections
*a* = 9.247 (2) Å	θ = 5–10°
*b* = 6.876 (1) Å	µ = 0.09 mm^−^^1^
*c* = 10.950 (2) Å	*T* = 293 K
β = 97.90 (3)°	Prism, colourless
*V* = 689.6 (2) Å^3^	0.75 × 0.53 × 0.20 mm
*Z* = 2	

### Data collection

**Table:**

Stoe Stadi-4 four-circle diffractometer	*R*_int_ = 0.0000
Radiation source: fine-focus sealed tube	θ_max_ = 26.0°, θ_min_ = 1.9°
graphite	*h* = −11→11
Scan width (ω) = 1.56–1.68, scan ratio 2θ:ω = 1.00 I(Net) and σ(I) calculated according to Blessing (1987)[Blessing, R. H. (1987). *Cryst. Rev.*1, 3–58]	*k* = 0→8
1483 measured reflections	*l* = 0→13
1479 independent reflections	3 standard reflections every 60 min
1000 reflections with *I* > 2σ(*I*)	intensity decay: 2%

### Refinement

**Table:**

Refinement on *F*^2^	Secondary atom site location: difference Fourier map
Least-squares matrix: full	Hydrogen site location: inferred from neighbouring sites
*R*[*F*^2^ > 2σ(*F*^2^)] = 0.064	H atoms treated by a mixture of independent and constrained refinement
*wR*(*F*^2^) = 0.144	*w* = 1/[σ^2^(*F*_o_^2^) + (0.034*P*)^2^ + 0.5511*P*] where *P* = (*F*_o_^2^ + 2*F*_c_^2^)/3
*S* = 1.15	(Δ/σ)_max_ < 0.001
1479 reflections	Δρ_max_ = 0.21 e Å^−^^3^
128 parameters	Δρ_min_ = −0.19 e Å^−^^3^
0 restraints	Extinction correction: *SHELXL*, Fc^*^=kFc[1+0.001xFc^2^λ^3^/sin(2θ)]^-1/4^
Primary atom site location: structure-invariant direct methods	Extinction coefficient: 0.009 (2)

### Special details

**Table:**

Geometry. All esds (except the esd in the dihedral angle between two l.s. planes) are estimated using the full covariance matrix. The cell esds are taken into account individually in the estimation of esds in distances, angles and torsion angles; correlations between esds in cell parameters are only used when they are defined by crystal symmetry. An approximate (isotropic) treatment of cell esds is used for estimating esds involving l.s. planes.
Refinement. Refinement of *F*^2^ against ALL reflections. The weighted *R*-factor wR and goodness of fit *S* are based on *F*^2^, conventional *R*-factors *R* are based on *F*, with *F* set to zero for negative *F*^2^. The threshold expression of *F*^2^ > σ(*F*^2^) is used only for calculating *R*-factors(gt) etc. and is not relevant to the choice of reflections for refinement. *R*-factors based on *F*^2^ are statistically about twice as large as those based on *F*, and *R*- factors based on ALL data will be even larger.

### Fractional atomic coordinates and isotropic or equivalent isotropic displacement parameters (Å^2^)

**Table:**

	*x*	*y*	*z*	*U*_iso_*/*U*_eq_	Occ. (<1)
O1	0.7289 (3)	0.2500	0.2635 (2)	0.0522 (8)	
O2	0.1375 (3)	0.2500	0.5696 (3)	0.0682 (10)	
N1	0.5853 (3)	0.2500	0.6240 (3)	0.0390 (8)	
C2	0.5055 (4)	0.2500	0.5163 (3)	0.0349 (9)	
N3	0.3551 (3)	0.2500	0.4988 (3)	0.0370 (8)	
C4	0.2711 (4)	0.2500	0.5924 (3)	0.0430 (10)	
C4A	0.3567 (4)	0.2500	0.7153 (3)	0.0384 (9)	
C5	0.2880 (4)	0.2500	0.8207 (4)	0.0493 (11)	
H5A	0.1865	0.2500	0.8133	0.059*	
C6	0.3684 (4)	0.2500	0.9343 (4)	0.0546 (12)	
H6A	0.3215	0.2500	1.0043	0.066*	
C7	0.5192 (5)	0.2500	0.9468 (4)	0.0531 (11)	
H7A	0.5731	0.2500	1.0251	0.064*	
C8	0.5901 (4)	0.2500	0.8445 (3)	0.0448 (10)	
H8A	0.6916	0.2500	0.8539	0.054*	
C8A	0.5102 (4)	0.2500	0.7258 (3)	0.0355 (9)	
C9	0.5544 (4)	0.2500	0.3964 (3)	0.0384 (9)	
C10	0.4244 (4)	0.2500	0.2975 (3)	0.0452 (10)	
H10A	0.4243	0.1352	0.2460	0.054*	0.50
H10B	0.4243	0.3648	0.2460	0.054*	0.50
C11	0.2918 (4)	0.2500	0.3684 (3)	0.0472 (10)	
H11A	0.2322	0.3648	0.3486	0.057*	0.50
H11B	0.2322	0.1352	0.3486	0.057*	0.50
C12	0.6934 (4)	0.2500	0.3777 (3)	0.0447 (10)	
H12A	0.7667	0.2500	0.4449	0.054*	
C13	0.8823 (4)	0.2500	0.2525 (4)	0.0586 (13)	
H13A	0.9291	0.1354	0.2916	0.070*	0.50
H13B	0.9291	0.3646	0.2916	0.070*	0.50
C14	0.8937 (5)	0.2500	0.1161 (4)	0.0673 (14)	
H14A	0.8438	0.1361	0.0788	0.081*	0.50
H14B	0.8438	0.3639	0.0788	0.081*	0.50
C15	1.0456 (5)	0.2500	0.0877 (5)	0.096 (2)	
H15A	1.0448	0.2500	−0.0001	0.144*	
H15B	1.0953	0.1360	0.1224	0.144*	0.50
H15C	1.0953	0.3640	0.1224	0.144*	0.50
O1W	0.9030 (4)	0.2500	0.7141 (4)	0.1047 (17)	
H2	0.975 (8)	0.2500	0.665 (6)	0.15 (3)*	
H1	0.816 (8)	0.2500	0.676 (7)	0.17 (3)*	

### Atomic displacement parameters (Å^2^)

**Table:**

	*U*^11^	*U*^22^	*U*^33^	*U*^12^	*U*^13^	*U*^23^
O1	0.0461 (16)	0.078 (2)	0.0357 (15)	0.000	0.0161 (12)	0.000
O2	0.0307 (15)	0.126 (3)	0.0479 (17)	0.000	0.0058 (12)	0.000
N1	0.0308 (16)	0.050 (2)	0.0367 (17)	0.000	0.0074 (13)	0.000
C2	0.0345 (18)	0.036 (2)	0.035 (2)	0.000	0.0094 (15)	0.000
N3	0.0347 (16)	0.045 (2)	0.0310 (16)	0.000	0.0030 (13)	0.000
C4	0.039 (2)	0.055 (3)	0.037 (2)	0.000	0.0115 (16)	0.000
C4A	0.0354 (19)	0.045 (2)	0.0363 (19)	0.000	0.0099 (15)	0.000
C5	0.037 (2)	0.070 (3)	0.043 (2)	0.000	0.0093 (17)	0.000
C6	0.049 (2)	0.082 (3)	0.037 (2)	0.000	0.0172 (18)	0.000
C7	0.055 (3)	0.070 (3)	0.034 (2)	0.000	0.0061 (18)	0.000
C8	0.038 (2)	0.061 (3)	0.036 (2)	0.000	0.0033 (16)	0.000
C8A	0.0328 (19)	0.039 (2)	0.036 (2)	0.000	0.0093 (15)	0.000
C9	0.043 (2)	0.041 (2)	0.0321 (19)	0.000	0.0089 (16)	0.000
C10	0.055 (2)	0.048 (3)	0.032 (2)	0.000	0.0055 (17)	0.000
C11	0.043 (2)	0.065 (3)	0.033 (2)	0.000	0.0015 (16)	0.000
C12	0.048 (2)	0.055 (3)	0.031 (2)	0.000	0.0086 (17)	0.000
C13	0.044 (2)	0.087 (4)	0.049 (2)	0.000	0.0206 (19)	0.000
C14	0.054 (3)	0.104 (4)	0.049 (3)	0.000	0.022 (2)	0.000
C15	0.063 (3)	0.164 (7)	0.063 (3)	0.000	0.020 (3)	0.000
O1W	0.046 (2)	0.204 (5)	0.065 (2)	0.000	0.0116 (19)	0.000

### Geometric parameters (Å, °)

**Table:**

O1—C12	1.336 (4)	C9—C12	1.329 (5)
O1—C13	1.439 (4)	C9—C10	1.502 (5)
O2—C4	1.226 (4)	C10—C11	1.539 (5)
N1—C2	1.302 (4)	C10—H10A	0.9700
N1—C8A	1.391 (4)	C10—H10B	0.9700
C2—N3	1.377 (4)	C11—H11A	0.9700
C2—C9	1.447 (5)	C11—H11B	0.9700
N3—C4	1.369 (4)	C12—H12A	0.9300
N3—C11	1.467 (4)	C13—C14	1.512 (5)
C4—C4A	1.464 (5)	C13—H13A	0.9700
C4A—C5	1.392 (5)	C13—H13B	0.9700
C4A—C8A	1.408 (5)	C14—C15	1.479 (6)
C5—C6	1.359 (5)	C14—H14A	0.9700
C5—H5A	0.9300	C14—H14B	0.9700
C6—C7	1.383 (5)	C15—H15A	0.9600
C6—H6A	0.9300	C15—H15B	0.9600
C7—C8	1.374 (5)	C15—H15C	0.9600
C7—H7A	0.9300	O1W—H2	0.91 (7)
C8—C8A	1.404 (5)	O1W—H1	0.85 (8)
C8—H8A	0.9300		
			
C12—O1—C13	116.8 (3)	C9—C10—H10A	110.8
C2—N1—C8A	116.3 (3)	C11—C10—H10A	110.8
N1—C2—N3	124.2 (3)	C9—C10—H10B	110.8
N1—C2—C9	127.8 (3)	C11—C10—H10B	110.8
N3—C2—C9	108.0 (3)	H10A—C10—H10B	108.9
C4—N3—C2	124.2 (3)	N3—C11—C10	104.6 (3)
C4—N3—C11	122.5 (3)	N3—C11—H11A	110.8
C2—N3—C11	113.3 (3)	C10—C11—H11A	110.8
O2—C4—N3	120.4 (4)	N3—C11—H11B	110.8
O2—C4—C4A	126.2 (3)	C10—C11—H11B	110.8
N3—C4—C4A	113.4 (3)	H11A—C11—H11B	108.9
C5—C4A—C8A	120.2 (3)	C9—C12—O1	120.8 (3)
C5—C4A—C4	120.7 (3)	C9—C12—H12A	119.6
C8A—C4A—C4	119.1 (3)	O1—C12—H12A	119.6
C6—C5—C4A	120.3 (4)	O1—C13—C14	106.6 (3)
C6—C5—H5A	119.9	O1—C13—H13A	110.4
C4A—C5—H5A	119.9	C14—C13—H13A	110.4
C5—C6—C7	120.6 (4)	O1—C13—H13B	110.4
C5—C6—H6A	119.7	C14—C13—H13B	110.4
C7—C6—H6A	119.7	H13A—C13—H13B	108.6
C8—C7—C6	120.5 (4)	C15—C14—C13	113.9 (4)
C8—C7—H7A	119.8	C15—C14—H14A	108.8
C6—C7—H7A	119.8	C13—C14—H14A	108.8
C7—C8—C8A	120.4 (4)	C15—C14—H14B	108.8
C7—C8—H8A	119.8	C13—C14—H14B	108.8
C8A—C8—H8A	119.8	H14A—C14—H14B	107.7
N1—C8A—C8	119.0 (3)	C14—C15—H15A	109.5
N1—C8A—C4A	122.9 (3)	C14—C15—H15B	109.5
C8—C8A—C4A	118.2 (3)	H15A—C15—H15B	109.5
C12—C9—C2	124.7 (3)	C14—C15—H15C	109.5
C12—C9—C10	125.7 (3)	H15A—C15—H15C	109.5
C2—C9—C10	109.6 (3)	H15B—C15—H15C	109.5
C9—C10—C11	104.5 (3)	H2—O1W—H1	115 (6)

### Hydrogen-bond geometry (Å, °)

**Table:**

*D*—H···*A*	*D*—H	H···*A*	*D*···*A*	*D*—H···*A*
O1w—H1···N1	0.85 (7)	2.13 (8)	2.968 (5)	165 (7)
O1w—H2···O2^i^	0.90 (7)	1.95 (7)	2.855 (5)	176 (6)

Symmetry codes:  (i) *x*+1, *y*, *z*.
